# Prostate Cancer in South Africa: Pathology Based National Cancer Registry Data (1986–2006) and Mortality Rates (1997–2009)

**DOI:** 10.1155/2014/419801

**Published:** 2014-05-15

**Authors:** Chantal Babb, Margaret Urban, Danuta Kielkowski, Patricia Kellett

**Affiliations:** ^1^NHLS/MRC Cancer Epidemiology Research Group (CERG), National Cancer Registry (NCR), National Health Laboratory Services (NHLS), Johannesburg 2000, South Africa; ^2^Faculty of Health Sciences, University of the Witwatersrand, Johannesburg 2000, South Africa

## Abstract

Prostate cancer is one of the most common male cancers globally; however little is known about prostate cancer in Africa. Incidence data for prostate cancer in South Africa (SA) from the pathology based National Cancer Registry (1986–2006) and data on mortality (1997–2009) from Statistics SA were analysed. World standard population denominators were used to calculate age specific incidence and mortality rates (ASIR and ASMR) using the direct method. Prostate cancer was the most common male cancer in all SA population groups (excluding basal cell carcinoma). There are large disparities in the ASIR between black, white, coloured, and Asian/Indian populations: 19, 65, 46, and 19 per 100 000, respectively, and ASMR was 11, 7, 52, and 6 per 100 000, respectively. Prostate cancer was the second leading cause of cancer death, accounting for around 13% of male deaths from a cancer. The average age at diagnosis was 68 years and 74 years at death. For SA the ASIR increased from 16.8 in 1986 to 30.8 in 2006, while the ASMR increased from 12.3 in 1997 to 16.7 in 2009. There has been a steady increase of incidence and mortality from prostate cancer in SA.

## 1. Introduction

Prostate cancer (ICD-O3 code C61.9 and ICD-10 code C61) (CaP) is one of the most common cancers worldwide. The worldwide incidence of CaP varies greatly between different geographical regions and/or ethnic groups, with men of African descent living out of Africa having some of the highest incidence rates (African American men 234.6 per 100 000) [1, 2]. Compared to Caucasian Americans, African Americans are disproportionately and more frequently diagnosed with CaP at an earlier age of onset, have higher tumour volume, more advanced (aggressive) tumour stage, higher Gleason score, and higher prostate specific antigen (PSA) levels [3, 4]. Indeed, there are differences in CaP mortality across men of different population groups; the mortality rate among African Americans (62.3 per 100 000) is 2.4 times the rate of Caucasian Americans (25.6 per 100 000) [1]. By contrast the incidence of CaP is low in several Asian countries [5]. The reasons for these differences are still unclear but may be related to differences in testing, referral patterns, access to care, differences in biology of the disease, inherited susceptibility, treatment options, reporting, and diagnosis; these could all influence disparities between different racial, ethnic, and geographic backgrounds [2, 6, 7].

Data from Africa on CaP is relatively sparse [2, 8]. The International Agency for Research on Cancer GLOBOCAN estimated 28 000 CaP deaths occurring in Africa in 2008 and predicted this number to double to 57 000 by 2030. There is belief of an underestimation of CaP in Africa as there may be a high degree of under diagnosis due to poor access to testing and diagnostic facilities [7, 9]. Globally, the incidence of CaP is increasing due to longer life spans, fewer deaths due to communicable diseases, increased PSA testing in the absence of symptoms, and as yet unknown aspects of westernization of lifestyle [10]. A better understanding of CaP rates in sub-Saharan Africa might provide valuable insight into the aetiology of CaP. The aim of this publication is to summarize data on CaP in South African (SA) men. Knowledge of cancer incidence is vital to inform health policy and effective service provision.

## 2. Methods

The primary source for the data was from the SA pathology based National Cancer Registry (NCR) reports from 1986 to 2006, from which the information on CaP was summarised by population group [11, 12]. The population groups are black, white, coloured (mixed ancestry), and Asian/Indian and reflect those used by the census data collected by the SA Government [13, 14]. The 2011 census indicated that 79.2% of the SA population were black, 8.9% white, 8.9% coloured, and 2.5% Asian/Indian. More detail was sourced directly from the NCR database and included a breakdown of the CaP subtypes seen through the years of 1999–2006 and the reported ages at diagnosis, allowing for calculation of mean ages of CaP cases reported to the NCR by population group. No information on stage or grade was available.

The NCR has been operating as a pathology based Cancer Registry with laboratory (microscopically, haematology, histology, and cytology) verified cases being reported and captured since 1986, with 2006 data being the latest year released [11, 12]. The NCR receives pathology reports from all pathology laboratories throughout the country with 84 countrywide laboratories reporting 80 000 confirmed cancer cases in 2010 [15]. New SA legislation (National Health Act-Regulations relating to cancer registration, 2011 Act No. 61 of 2006 No. R. 380) is now shifting the registry to a population based surveillance system [16].

Data submitted is coded according to the International Classification for Oncology, third edition (ICD-O3), by trained staff [15, 17, 18]. It is now common practice for the NCR to receive full pathology reports, including clinical details, which makes for more accurate cancer coding and database completeness. When possible, past NCR data are constantly updated with newly developed and improved quality control measures, such as the conversion of data from other coding formats to ICD-O, as well as checking for incorrect/improbable coding [18]. Reporting is done in ICD-10 coding to allow for comparison with global registry reports.

The use of age standardised rates (ASR) is necessary when comparing several populations that have different age compositions. Age standardisation for NCR by population group was done using mid-year estimates for the SA population from Dorrington et al. [19] to provide crude estimates and the 1960 world standard population [20] to per 100 000.

In addition, data from Statistics SA on mortality by malignancy for the SA population (1997–2009) was made available [21, 22]. From this data age standardised mortality rates were calculated using the direct method with the same mid-year populations used for the NCR [19, 20]. Statistics SA reports mortality on a national level [21] and not by population group but, on request from NCR, reporting of cancer mortality by population group was made available for the year 2009 only.

## 3. Results

### 3.1. Incidence

**Figure 1:**
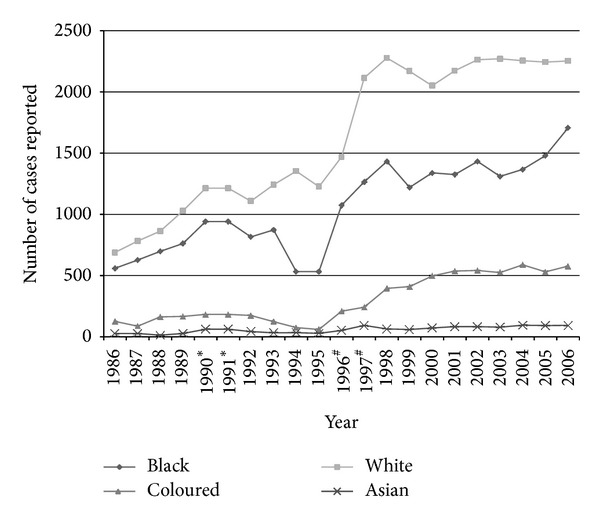
Number of prostate cancer cases reported to the South African pathology based National Cancer Registry (NCR) from 1986 to 2006 by the population groups; white, black, coloured, and Asian/Indian. ^#^1996 and 1997 coloured and whites were pooled together in the published report but NCR provided the number of cases per year. *Years 1990-1991 were combined and reflect the average number of reported cases over the two years.

In 1995, 2504 new cases of CaP were reported and 10 years later, in 2006, there were 4631 new cases (Table 1). Over the 20-year time period, 1986 to 2006, 63 886 CaP were reported to NCR. Each year the white population group had the highest number of cases reported. Both the coloured and Asian/Indian population groups have relatively small numbers, with less than a hundred cases reported each year for the Asian/Indian population. Both the black and white population groups have fairly consistent numbers being reported through the years, in particular for the last 5 years of reporting. There is a trend for a steady increase in the number of CaP cases reported in each population group through the years. The calculated year-on-year percentage change (data not shown) in CaP cases showed that in the period 1986 to 1995 there was quite a large variation in the different population groups (from +129.6% to minus 39.5%) although for all groups combined it ranged from +19% to minus 4.5%. The most dramatic increase occurred between 1996 and 1999 (Figure 1). In the period 1996-97 there was a substantial increase (+12% to +33%). This increase was the largest in the Asian/Indian and white/coloured populations but also occurred in the black group. After 1999 the annual percentage change in CaP numbers decreased to low levels (+6.6% to minus 7.5%). Nearly all (96%) of the CaPs reported to the NCR from 1999 to 2006 were adenocarcinoma, followed by unspecified carcinomas (3%).

CaP was ranked as the number one cancer in SA men since 1996 (excluding basal cell carcinoma). Prior to this CaP was either second to oesophageal cancer in the black population (1990–1995) or lung cancer in the coloured population (1988 and 1989).

**Figure 2:**
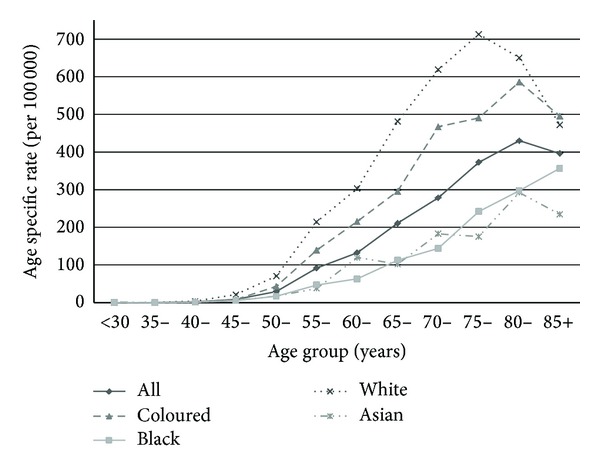
South African pathology based National Cancer Registry age specific incidence rate (ASIR) per 100 000 for prostate cancer, 2006, by population group.

CaP occurs in men over the age of 45 years and rates steadily increase with age (Figure 2). The mean age of men with CaP reported to the NCR in 2006 was 68 years (standard deviation (SD) 9.6); for black men it was 68 years (SD 10.5), for white men it was 67 years (SD 9.0), for coloured men it was 67 years (SD 9.2), and for Asian/Indian men it was 66 years (SD 10.1). From 1986 to 2006 3.6% of men with CaP were younger than 50 years of age and 37.6% were younger than 65.

**Figure 3:**
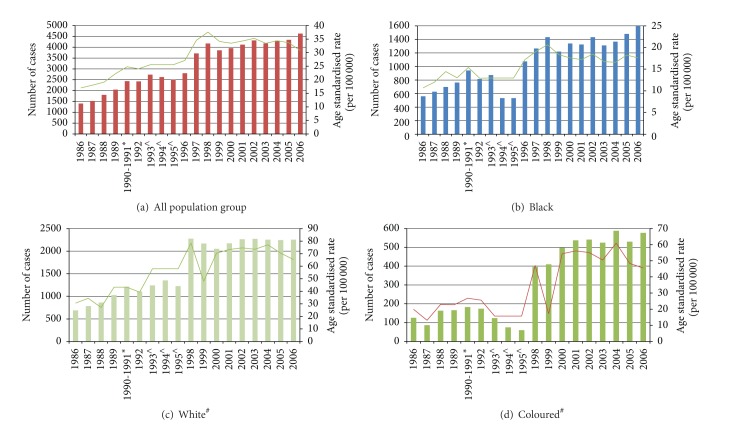
South African pathology based National Cancer Registry reported incidence of prostate cancer, 1988–2006. Columns: number of cases. Line: age standardised incidence rate (ASR). (a) All population groups; (b) black; (c) white; (d) coloured. ^∧^1993–1995 data was combined and the ASR is for the 3-year period. ^#^1996 and 1997 coloured and whites were pooled together and cannot be reported separately. *Years 1990-1991 were combined and reflect the average number of reported cases over the two years. Asian/Indian population had less than 100 cases so not included here.

The ASR for CaP in SA was 17 per 100 000 in 1986, increasing steadily and by 2006 was 27 per 100 000 (Table 2). The black population had the lowest ASR of 12 per 100 000 compared to 52 per 100 000 in white men (Table 2 and Figure 3).

### 3.2. Mortality

CaP accounted for 1670 deaths in SA in 1999, 1954 deaths in 2004, and 2331 deaths in 2009 indicating a steady increase (Table 3).

In SA men from 1997 to 2005, CaP was the third leading cause of death through a malignancy (10%). In 2004 the most frequent cause of death through a malignancy in SA men was lung cancer (17.7%) followed by oesophageal cancer (12.6%). In 2006 CaP (12% of deaths by a malignancy) overtook oesophageal cancer (10.7%) to become the second most common cause of death through a malignancy. In 2009 lung cancer was still the most common cause of death by a malignancy in men (19%), followed by CaP (13%) and then oesophageal cancer (11%).

The mean age of men who died from CaP in 2009 was 74 years (SD 10.6); for black men it was 73 years (SD 10.8), for white men it was 72 years (SD 10.4), for coloured men it was 76 years (SD 10.1), and for Asian/Indian men it was 76 years (SD 9.1). Thirteen percent of malignancy deaths in men were due to CaP, with 78% being older than 65 years.

In SA the age standardised mortality rate in 2009 for CaP was 16.7 per 100 000 and in 1997 it was 12.3 (Table 3). By population group the age standardised mortality rate was 11.4 for black men, 6.8 for white men, 51.9 for coloured men, and 6.3 for Asian/Indian men (Table 4).

### 3.3. Incidence and Mortality

**Figure 4:**
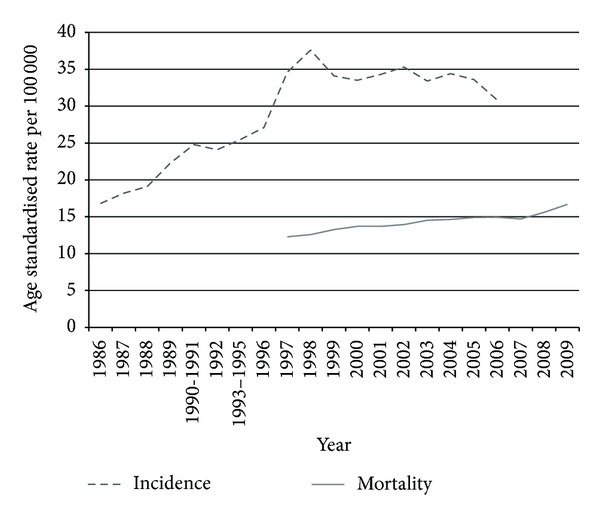
Prostate cancer in South Africa, age standardised incidence rate (1986–2006) from the pathology based National Cancer Registry and age standardised mortality (1997–2009) rate from Statistics SA mortality data, per 100 000.

The proportion of the reported male cancers attributed to CaP increased from 7% in 1986 to 17% in 2006 (Table 2). Almost half of the reported CaP cases were from the white population (Table 1) who represent 9% of the SA population [13]. Of the 2331 reported deaths from CaP in 2009, 971 (42%) were black, 529 (23%) were from unknown population group, 546 (22%) were coloured, 251 (11%) were white, and 34 (2%) were Asian/Indian. White men represent a higher proportion of cases in the NCR (49%) but a smaller proportion of deaths from CaP (14% versus 49%, resp.), compared to black men who represent 37% of CaP yet 54% of the deaths from CaP (Table 4). The age standardised rates for incidence (1986–2006) and mortality (1997–2009) have been increasing through the years (Figure 4).

## 4. Discussion

The first published NCR report was in 1986 with a total of 35 569 cancers reported in a total population of just under 29.3 million. In 2006, 55 241 new cancer cases were reported to the NCR in a total population under 47.5 million people. Since 1986 the cancer burden increased by 64% while the population increased by 62%, a minor increase in cancer.

As SA has a pathology based registry, CaP cases that were diagnosed without a biopsy but with a digital rectal examination (DRE) and PSA [7] would not be captured [23]. Thus, the true rate of CaP in SA men is higher than the data indicates.

**Figure 5:**
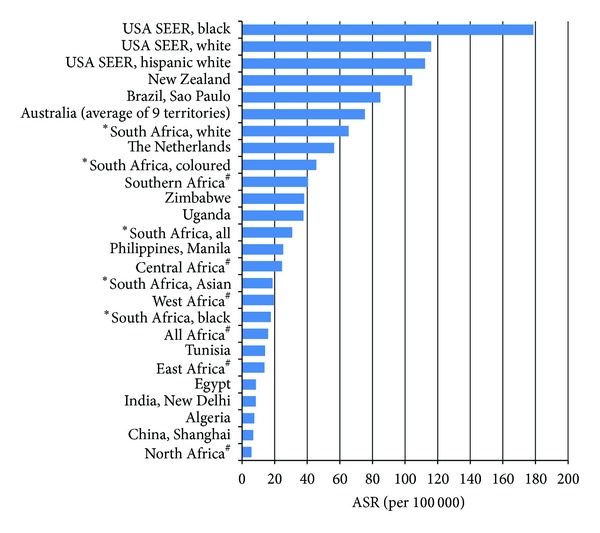
Comparison of age standardised rates (ASR) per 100 000 for prostate cancer from Curado et al. 1998–2002 [5], Parkin et al. 2002 (#) [37], and South African pathology based National Cancer Registry 2006 (∗).

A rural/regional population based registry (PROMEC) based in the Eastern Cape Province of SA reported an ASR of 4.4 per 100 000 for CaP in 1998–2002 [24], versus the 17 per 100 000 from the NCR data for the SA black population. This could be a reflection of poor access to services in the Eastern Cape but it may also reflect different environmental exposures in this rural setting and may be a consequence of low prevalence rather than failure to diagnose and register cases [7]. However, the Eastern Cape has a large migratory population and men in particular may relocate to larger cities seeking work. These men may ultimately be diagnosed and treated in urban areas and would not reflect in the PROMEC registry [25]. Compared to other sub-Saharan African countries, our data does report a lower rate of CaP for the black population (Figure 5). For example in Zimbabwe, a neighbouring country with a population based registry, the ASR is 38.1 per 100 000 (1998–2002) [5]. It is highly likely that there is an underestimation of incidence from NCR data.

As cancer and in particular CaP is a disease of age we must consider the age distribution of the population with a number of competing causes of mortality in the various SA population groups. It also needs to be noted that there is a massive age structure difference between the SA population groups. SA has an intermediate aged population with an average age of 25 years: 21 years for the black population, 26 years for the coloured population, 32 years for the Asian/Indian population, and 38 years for the white population [13]. In 2004 the life expectancy at birth was 51.4 years for the total SA population, for black men it was 47.8 years whereas for white men it was 61.7 years [26]. By 2011 the life expectancy at birth for SA men increased to 54.9 years [27]. Despite this, there is little variation in age at diagnosis between the different population groups, all being in the region of 68 years old at diagnosis and 74 years old at death. However, the cancer stage at presentation is unknown, and therefore we cannot determine if any possible lead time bias occurred in any particular population group. From 1986 to 2006, 3.6% were younger than 50 years of age and 37.6% were younger than 65 (Figure 2); this is far more than the reported percentage of <0.1% of all patients and about 85% being diagnosed after the age of 65 [28]. In the UK 12% of malignancy deaths in men were due to CaP, with 93% older than 65 years [29]. This may however, again, reflect the age structure of the SA population.

Some points to consider about NCR reports are only pathology based cases reported; the 1990-1991 NCR report has summarised data reported for the two years as one. The 1993 to 1995 NCR report is similar, with summarised data reported for all 3 years as one, but the numbers of observed cases are given per year. The 1996-1997 NCR report has the coloured and white population groups pooled together; however the NCR was able to provide the reported numbers reported for each group separately. Since 1996 the observed numbers of cases were adjusted to take into consideration the unknowns (cases reported with no age or undetermined population). From 1998 there were positive changes in the NCR data coding, capturing protocols, and the detection of duplicates, improving the quality of data.

The incidence of CaP is influenced largely by testing and, where testing is common practice with subsequent biopsy, CaP rates are likely to increase. As a result, countries that implemented PSA testing, many in the 1990s, saw an increase of CaP after the introduction, followed by a decrease and then stabilisation of CaP numbers a few years later [10]. Globally, several trials to determine effectiveness of PSA testing have been done with no conclusive resolution of effectiveness and the cost benefit in asymptomatic men [30, 31]. There is concern around PSA testing in that it may result in over diagnosis [10]. PSA testing does have value in diagnosing symptomatic early stage CaP and for monitoring treatment response [28]. However, a test capable of determining the risk of having CaP with rapid progression is lacking and there is a need to identify life-threatening CaP and potential progression of disease.

In SA, the period of introduction and the frequency of testing with PSA are unclear. But in the mid-90s PSA testing did become more popular and was more frequently used in diagnosis of CaP (personal communication). The NCR data does indicate that there was a sudden increase in the number of CaP cases across all population groups (Table 1 and Figure 1) and this increase in the mid-90s was not seen with other cancers in the NCR reports. It is highly probable that the introduction of PSA testing was responsible for the increase of CaP cases being diagnosed from 1996 to 1999 (Figure 1).

PSA testing in asymptomatic men can be considered not to be readily available in the SA public health care sector and most men will present with symptoms before being tested [32]. However, it may be more readily available in the SA private health care sector. Having medical aid cover allows for access to private healthcare facilities. In 2011, 69.7% of the white population belonged to a medical scheme, with 41.1% of the Asian/Indian population, 20.3% of the coloured population, and 8.9% of the black population [33]. This may explain the incidence versus mortality rate seen in white men (Table 4); as they are likely to belong to a medical aid scheme they have access to a private health care facility that will, possibly, more readily provide PSA testing. These men may get diagnosed earlier and receive life-saving treatment or potentially unnecessary treatment. In the UK, where the health care system is free and has less variation in quality of care by socioeconomic status, a study comparing black men with white men found, apart from early age of onset in black men, that there was no evidence of differences in disease characteristics at the time of CaP diagnosis or of under-investigation or under treatment in UK black men compared to UK white [9]. It may be that in SA differential detection by population group is due to differences in socioeconomic characteristics such as access to and use of health care facilities [14, 28]; this is one of many possible factors.

Heyns et al. in the Western Cape Province of SA looked at PSA use in their public health care centre. They found a significant problem in getting men with an elevated serum PSA (>4 ng/mL) to undergo a prostate biopsy, with uptake ranging from 19% to 47% in the different population groups, black and coloured, respectively [23]. Although an earlier, unpublished study by the same centre experienced uptake (when biopsy was indicated) between 53% and 87%, for black and white / mixed ancestry, respectively [23]. This is one center's experience and the ages of the nonbiopsied were not provided (older men may not have wanted to be biopsied due to quality of life concerns). However, if the situation is similar throughout SA, it could also contribute to underestimation of CaP when a pathology based registry is in operation. Further studies are required to determine to what extent uptake for a biopsy after an elevated PSA is detected, why it is occurring, and how uptake for a biopsy could be improved.

The 2011 census indicated that 79.2% of the SA population were black, 8.9% white, 8.9% coloured, and 2.5% Asian/Indian. The incidence and mortality rates do not appear to reflect the demographics of the country (Table 4). Additional research is required to identify these issues and develop interventions that will minimize these effects.

Compared to incidence, mortality is less often affected by diagnostic and screening practices but reflect differences in CaP treatment worldwide, as well as underlying risk [10]. GLOBOCAN (2008) reported an overall mortality rate for Southern Africa as 19.3 (per 100 000), 9.9 for N. America and 12 for Europe [34]. In SA the age standardised mortality rate in 2008 for CaP was 15.7 per 100 000; in 1997 it was 12.3 (Table 3). By population group the age standardised mortality rate was 11.4 for black men, 6.8 for white men, 51.9 for coloured men, and 6.3 for Asian/Indian men (Table 4). Coloured SA men have the highest mortality rate.

Awareness campaigns on men's health, such as Movember [35] and other similar campaigns should be further developed, testing drives and improved education around men's health; CaP and improved follow-up of potential patients that have increased PSA levels are all required. Other risk factors such as healthcare access, lifestyle, medical care beliefs/practices, environmental exposures, or biological (including genetic) mechanisms may also be influencing the disparities between the populations and need to be further investigated. In addition the influence of HIV and its management on risk of CaP is also still unknown and needs to be explored [36].

## 5. Conclusion

CaP is a growing problem in SA and globally; it is the most common cancer in men and the second most common malignancy causing death for men. We acknowledge that SA NCR CaP incidence rates are an underestimate of the total burden of this cancer in the country, due to the fact that the NCR records only microscopically verified cases and that not all geographic areas and sociodemographic groups have equal access to all tiers of health services. In spite of these limitations the NCR data, combined with the national cancer-specific mortality data, do provide insight into the demographics of CaP in SA as they give minimum incidence and rates. The results give an indication of the bare minimum numbers of CaP cases that have been diagnosed and which need to be accommodated in the SA health care system. As well as possible genetic influences, lifestyle and health care access will account for some of the differences between population groups and it is possibly an indication that reporting and diagnostics for CaP are not adequate in SA. It is also anticipated that there will be an increase of CaP in SA as lifestyles change and the SA population advances in age with the life expectance rising. A better understanding of the burden of CaP in SA, as well as studies to determine the appropriate care programs required, ensuring adequate and appropriate diagnosis and treatment in all sectors are needed. The data presented here is the only consolidated data available for SA. Despite the limitations of the data it provides information for researchers, clinicians, policy makers, NGOs, and other stakeholders working with men's health and CaP.

There is a need for improvements in awareness, diagnosis, and treatment of CaP. Future expansion of the SA NCR to include hospital and population based components will assist in tracking changes. A better understanding of the aetiology and underlying biological mechanisms to modifiable risk factors of CaP across all populations can potentially improve the care of all CaP patients everywhere and could have implications for selective testing, prevention, and treatment options, ensuring valuable resources are appropriately utilized.

## Figures and Tables

**Table 1:** The number of prostate cancer cases reported to the South African pathology based National Cancer Registry, years 1986–2006, by population group.

	All	Black	White	Coloured	Asian
1986	1401	559	689	126	27
1987	1522	627	782	86	27
1988	1800	698	863	163	13
1989	2046	762	1028	166	27
1990-1991	2434	941	1213	183	62
1992	2424	816	1108	174	43
1993	2736	873	1241	124	33
1994	2622	532	1353	75	34
1995	2504	532	1226	60	28
1996*	2802	1074	1468^∧^	209^∧^	52
1997*	3715	1265	2115^∧^	241^∧^	93
1998*	4171	1432	2277	396	64
1999*	3860	1220	2169	410	59
2000*	3958	1339	2051	497	72
2001*	4118	1325	2173	537	83
2002*	4318	1432	2263	541	83
2003*	4178	1310	2271	525	79
2004*	4301	1367	2255	588	94
2005*	4346	1480	2244	530	92
2006*	4631	1707	2253	577	93

Years 1986–1995 reported observed numbers and years *1996–2006 reported adjusted numbers. ^∧^In 1996 and 1997 whites and coloureds were reported together; NCR provided the individual figures that are not in the printed report.

**Table 2:** South African pathology based National Cancer Registry summary statistics for prostate cancer years 1986–2005 by population group.

	Life time risk (0–74) (LR)						Age standardised rates (ASR)						Proportion (%)				
	All	Black	White	Coloured	Asian		All	Black	White	Coloured	Asian		All	Black	White	Coloured	Asian
1986	NR	NR	NR	NR	NR		16.8	10.7	30.6	19.9	15.9		7.37	8.15	6.56	9.43	8.82
1987	NR	NR	NR	NR	NR		18.2	12.0	34.4	13.1	17.3		9.18	10.07	8.47	8.99	12.98
1988	1 in 44	1 in 52	1 in 32	1 in 38	1 in 173		19.1	14.4	27.1	23.0	6.5		7.68	8.00	7.11	9.12	4.56
1989	1 in 39	1 in 64	1 in 21	1 in 40	1 in 52		22.3	13.0	43.3	22.8	14.3		8.14	8.21	8.26	9.51	7.34
1990-1991*	1 in 32	1 in 49	1 in 19	1 in 29	1 in 60		24.8	15.4	43.3	26.8	13.5		8.68	8.80	8.46	11.23	4.21
1992	1 in 33	1 in 58	1 in 20	1 in 35	1 in 67		24.1	12.9	39.3	25.6	11.7		9.36	9.00	8.97	12.11	10.02
1993–1995*	1 in 31	1 in 61	1 in 14	1 in 50	1 in 47		25.5	13.0	58.0	15.8	17.6		10.91	10.43	10.86	12.23	8.23
1996^†^	1 in 31	1 in 50	1 in 18		1 in 39		27.1	17.2	45.7		18.9		11.10	11.48	10.97		8.86
1997^†^	1 in 24	1 in 47	1 in 23		1 in 29		34.6	19.1	37.9		32.4		12.59	12.49	7.91		12.99
1998	1 in 22	1 in 42	1 in 10	1 in 17	1 in 46		37.6	20.6	78.5	47.1	20.4		14.14	13.41	14.59	15.88	11.09
1999	1 in 24	1 in 39	1 in 19	1 in 50	1 in 39		34.1	18.3	48.0	17.2	18.3		13.12	10.85	14.66	12.42	10.85
2000	1 in 24	1 in 47	1 in 11	1 in 15	1 in 38		33.5	17.6	70.4	54.4	23.0		14.01	14.45	13.62	14.96	12.01
2001	1 in 23	1 in 48	1 in 11	1 in 14	1 in 34		34.3	17.2	73.4	56.1	22.9		13.80	13.97	13.41	15.17	13.27
2002	1 in 23	1 in 46	1 in 11	1 in 15	1 in 44		35.3	18.5	74.7	55.1	22.2		15.35	15.70	14.85	16.98	14.30
2003	1 in 24	1 in 49	1 in 11	1 in 17	1 in 42		33.4	16.7	73.5	50.4	18.2		14.87	15.44	14.48	15.83	12.82
2004	1 in 23	1 in 48	1 in 10	1 in 13	1 in 34		34.4	16.6	77.1	60.9	23.2		15.63	16.04	15.10	17.03	15.28
2005	1 in 24	1 in 48	1 in 14	1 in 16	1 in 35		33.7	18.2	70.5	48.4	23.0		16.84	17.12	16.92	16.70	15.46
2006	1 in 27	1 in 52	1 in 12	1 in 18	1 in 43		30.8	17.6	65.4	45.5	18.8		17.28	17.87	16.94	17.61	13.81

*Summary statistics for the years 1990-1991 (2 years) together, 1993–1995 (3 years) together, and ^†^white and coloured population groups were pooled. NR: not reported. Asian is the Asian/Indian population group.

**Table 3:** Summary information for statistics South Africa mortality, from 1997 to 2009, ICD-10 code C61, prostate cancer.

Year	Number of reported deaths from Prostate Cancer	Proportion (%) of all reported male deaths by a malignancy	Crude rate for men	Age standardised mortality rate for men
1997	1498	9.38	7.23	12.29
1998	1553	9.26	7.37	12.59
1999	1670	9.71	7.80	13.27
2000	1752	10.18	8.06	13.71
2001	1779	10.38	8.07	13.71
2002	1819	10.21	8.15	13.95
2003	1914	10.56	8.48	14.53
2004	1954	10.57	8.58	14.65
2005	2005	11.09	8.72	14.90
2006	2160	12.00	9.26	14.94
2007	2216	11.95	9.40	14.71
2008	2317	12.67	9.88	15.60
2009	2331	13.30	9.77	16.67

**Table 4:** Prostate cancer in South Africa, comparison of 2009 mortality data by population group (when population group was known) to 2006 cancer incidence of reported cases to the pathology based National Cancer Registry (latest report available).

	2009 mortality			2006 cancer incidence		
	Total number of cases	%	Age standardized mortality rate	Total number of cases (adjusted)	%	Age standardized incidence rate
All	2331	—	16.7	4631	—	30.8
Black	970	54	11.4	1707	37	17.6
White	251	14	6.8	2253	49	65.4
Coloured	546	30	51.9	577	12	45.5
Asian	34	2	6.2	93	2	18.8
